# Crossing classroom boundaries: school-based music participation and cross-domain learning development in adolescence

**DOI:** 10.3389/fpsyg.2026.1975580

**Published:** 2026-10-07

**Authors:** Xiaoyu Peng, Xin Shan, Mengqi Zhang, Huasen Zhang, Kangrui Sun

**Affiliations:** 1 The Graduate School Arts and Culture, Sangmyung University, Seoul, Republic of Korea; 2 The Graduate School & Education, Sangmyung University, Seoul, Republic of Korea

**Keywords:** adolescent learning development, classroom climate, learning engagement, mathematics self-efficacy, music education, PISA 2022, school-based music participation

## Abstract

**Background:**

In education systems where curriculum time is limited and core academic subjects are strongly prioritized, a key question is whether music participation merely entails opportunity costs in instructional time or is also associated with learning and psychological resources that can be mobilized across disciplinary contexts.

**Objective:**

This study examined whether school-based music participation is associated with domain-general learning engagement and adolescent learning development in a core academic subject; given mathematics’ cognitive demands and salient evaluative and emotional processes, mathematics was selected as a representative domain.

**Methods:**

Using the Korean PISA 2022 sample (*N* = 6,454; 5,914 with valid music-participation data), survey-weighted models incorporated replicate weights and mathematics plausible values and tested indirect and conditional pathways involving mathematics self-efficacy, anxiety, teacher support, disciplinary climate, and achievement.

**Results:**

More frequent school-based music participation was positively associated with all eight engagement indicators in the primary analyses (*β* = 0.034–0.083). Domain-general engagement was indirectly associated with mathematics achievement primarily through mathematics self-efficacy (*B* = 0.498, *p* < 0.001), whereas the overall mathematics-anxiety pathway was nonsignificant. Exploratory analyses suggested that MATHPERS may serve as a behavioral bridge. Teacher-support moderation was concentrated in mathematics anxiety, but neither classroom condition altered the overall cross-domain indirect association.

**Conclusion:**

School-based music participation was associated with multidimensional resources relevant to adolescent learning development, supporting a resource-formation and domain-specific recontextualization account and suggesting that music education should be evaluated not only by direct achievement transfer but also by its broader contribution to learning resources across subjects.

## 1 Introduction

In contemporary education, whether students can apply knowledge, competencies, and psychological resources developed in one context to new tasks has become an important concern in curriculum reform. The OECD future education framework similarly emphasizes students’ capacity to mobilize integrated knowledge, skills, attitudes, and values in complex and unfamiliar situations (OECD, 2019). STEAM education and UNESCO’s *Framework for Culture and Arts Education* reflect this broader trend and highlight the role of culture and the arts in holistic and cross-domain education (UNESCO, 2024).

However, curriculum time and educational resources remain limited, particularly in systems dominated by high-stakes subjects such as literacy and mathematics. Arts subjects, including music, may consequently face reductions in curriculum time and resources (Browne, 2022; UNESCO, 2024). Given that musical activities simultaneously involve cognitive engagement, learning motivation, emotion regulation, and social interaction, a central question arises: does greater participation in the arts primarily reduce time available for core academic subjects, or is it also associated with learning and psychological resources that can be mobilized across contexts?

Music education provides a representative context in which to examine this question. Music learning involves not only artistic skills but also sustained attention, persistent practice, feedback-based adjustment, and social interaction. Previous research has linked music participation with general cognitive functioning, learning engagement, emotional functioning, and academic performance, although the magnitude and robustness of these associations vary across activity types, instructional structures, cultural contexts, research designs, and outcome measures (Bigand and Tillmann, 2022; Blasco-Magraner et al., 2021; Ilari and Cho, 2023; Román-Caballero et al., 2022; Wang et al., 2026). Examining only direct associations between music participation and distal outcomes therefore provides an incomplete account of its potential cross-domain mechanisms.

Rather than treating such associations as direct transfers of ability, it may be more informative to consider how resources are transformed across learning contexts. Domain-general resources developed or reinforced through musical practice—such as sustained engagement, active exploration, learning motivation, and social–emotional connectedness—may be carried into new disciplinary contexts. Transfer research similarly suggests that transfer is not a simple replication of existing abilities but depends on the relation between prior learning, task demands, and the new context (Barnett and Ceci, 2002; Bransford and Schwartz, 1999). Once students enter a target subject, these resources may be reorganized in relation to its task structures, evaluative demands, and emotional experiences, consistent with situated accounts of transfer that emphasize how learners understand and participate in new activity contexts (Lobato, 2006). Through this process of domain-specific recontextualization, domain-general resources may become expressed as domain-specific competence beliefs, emotional states, and learning behaviors, which in turn become associated with observable academic outcomes. Music education’s cross-domain role can therefore be conceptualized as a theoretical sequence of resource formation–cross-context mobilization–domain-specific recontextualization–academic manifestation. In the present study, this sequence represents a theoretically ordered pattern of associations rather than an observed developmental progression over time.

Ecological systems theory provides a further theoretical basis for this perspective. Bronfenbrenner (1979) proposed that individuals are simultaneously embedded in multiple interconnected immediate contexts and that the relationships among microsystems are themselves important for understanding development. Building on this logic, rather than treating the school or classroom as a single homogeneous context, the present study analytically distinguishes subject-specific learning environments within the school. Music, mathematics, and other subjects expose students to partly different task structures, evaluative demands, instructional practices, and social interactions. The proposed heterogeneous parallel disciplinary microsystems perspective therefore represents an extension of Bronfenbrenner’s microsystem logic to differentiated disciplinary contexts within schooling, rather than a concept directly specified in the original theory. Different school subjects are conceptualized as relatively distinct learning microsystems that nevertheless become connected through the student. Students may function as carriers of resources, bringing domain-general resources developed or reinforced in one disciplinary context into another and further recontextualizing those resources in relation to new tasks and classroom conditions.

Within this framework, mathematics is used as a representative dimension of adolescent learning development within a core academic domain and as the target context in which cross-domain resource transformation is examined. Mathematics learning places substantial demands on logical reasoning and problem solving (OECD, 2023) while also carrying explicit evaluative demands. It is therefore especially likely to elicit domain-specific competence judgments and emotional responses, including mathematics self-efficacy and mathematics anxiety (Bandura, 1997; Barroso et al., 2021; Pekrun, 2006). Mathematics achievement also provides a clear and comparable indicator of academic performance. Accordingly, the present study treats mathematics achievement as a representative observable manifestation of adolescent learning development within a specific core academic domain, allowing examination of how domain-general learning resources may undergo mathematics-specific psychological recontextualization and become associated with observable academic performance.

The present study examines this process using the Korean sample from PISA 2022. Korea has a relatively well-established school arts-education context alongside high levels of academic performance, providing a useful setting in which to examine relationships between arts participation and learning in core academic subjects. More importantly, mathematics was the major assessment domain in PISA 2022, and the dataset simultaneously includes information on school-based music participation, multidimensional learning engagement, mathematics self-efficacy and anxiety, mathematics teacher support, disciplinary climate, and mathematics achievement. This enables resource formation, domain-specific psychological recontextualization, and relevant classroom conditions to be examined within the same large-scale data framework.

In summary, the present study neither assumes that music participation necessarily improves mathematics achievement nor dismisses the potential opportunity costs associated with curriculum time for the arts. Instead, it examines whether school-based music participation is associated with domain-general learning resources that can be mobilized across contexts, and whether these resources, once situated within mathematics as a specific core academic domain, are further associated with mathematics achievement through domain-specific psychological processes. The following research questions and hypotheses were addressed:

RQ1: Is the frequency of school-based music participation associated with adolescents’ domain-general learning engagement (behavioral engagement, cognitive engagement, and emotional–motivational engagement)?

RQ2: Is school-based music participation serially indirectly associated with mathematics achievement through domain-general learning engagement and its domain-specific recontextualization into mathematics self-efficacy and mathematics anxiety?

RQ3: Does the mathematics classroom environment moderate the associations between domain-general learning engagement and mathematics self-efficacy and anxiety, and thereby influence the indirect association between music participation and mathematics achievement?

RQ3a: Does mathematics teacher support moderate the associations between domain-general learning engagement and mathematics self-efficacy and anxiety, and thereby influence the broader cross-domain indirect association?

RQ3b: Does mathematics disciplinary climate moderate the associations between domain-general learning engagement and mathematics self-efficacy and anxiety, and thereby influence the broader cross-domain indirect association?

## 2 Literature review

### 2.1 From music participation to domain-general learning-engagement resources

Learning engagement is a multidimensional state in which students continuously mobilize behavioral, cognitive, and emotional resources during learning, and is commonly conceptualized in terms of behavioral, cognitive, and emotional dimensions (Fredricks et al., 2004). In the present study, this framework is operationalized as behavioral engagement, cognitive engagement, and emotional–motivational engagement, representing domain-general resources that may function across learning contexts. Importantly, these dimensions are not treated as interchangeable indicators of a single latent construct. Instead, “domain-general learning engagement” serves as an organizing framework for distinct but complementary engagement-related resources, with the available PISA indicators interpreted as proxies rather than as measures of a standardized engagement scale. Music learning involves all three forms of engagement.

Performance, singing, and rehearsal typically require sustained practice, performance monitoring, and behavioral adjustment in response to feedback, placing substantial demands on persistence and self-regulation (Evans, 2023; Nusseck and Spahn, 2021; Vellacott and Ballantyne, 2022). Experimental and review evidence also suggests positive associations between music learning and general cognitive functions such as inhibitory control, working memory, and cognitive flexibility, although effects vary across cognitive functions and study designs (Frischen et al., 2021; Jamey et al., 2024; Lu et al., 2025).

Beyond behavioral and cognitive resources, musical activities also involve substantial emotional and social engagement. Ilari and Cho (2023) found that music participation among secondary-school students was associated with school connectedness and positive youth development, while a systematic review reported positive associations between educational uses of music and children’s emotional recognition, expression, and regulation (Blasco-Magraner et al., 2021). Research with Korean adolescents further suggests that music listening may be associated with lower everyday stress responses through concentration and mood regulation (Kim, 2024). Because that study focused specifically on music listening rather than broader forms of music participation, it is used here only as supplementary evidence linking musical experience with self-regulatory resources.

Music participation may therefore be associated not only with musical skills but also with domain-general resources—such as persistence, active exploration, learning motivation, and school connectedness—that can potentially be mobilized across learning contexts. Accordingly:

*H1*: More frequent participation in school-based music classes or activities is positively associated with adolescents’ domain-general learning engagement.

### 2.2 From domain-general learning-engagement resources to domain-specific psychological resources

Domain-general learning-engagement resources are not equivalent to competence judgments or emotional experiences within a specific academic subject. A student may show high persistence, exploratory tendencies, or school belonging, yet these general resources still need to acquire subject-specific meaning when applied to particular disciplinary tasks. According to Bandura’s (1997) theory of self-efficacy and Pekrun’s (2006) control–value theory, self-efficacy and achievement emotions are strongly task- and context-specific. Recent evidence also indicates that broader school resources and domain-specific academic outcomes are linked through psychological processes. For example, school belonging is closely associated with engagement and broader psychological adjustment (Allen et al., 2024; St-Amand et al., 2021), whereas mathematics self-efficacy is related to behavioral engagement and mathematics achievement (Shimizu, 2025). Thus, domain-general effort, persistence, and positive engagement may need to become expressed as domain-specific competence beliefs and emotional states after entering mathematics learning before they develop more proximal associations with mathematics outcomes.

On the one hand, mathematics self-efficacy reflects students’ beliefs about their capability to complete mathematics tasks. Whether general persistence and cognitive engagement become translated into a belief that “I can successfully complete this mathematics task” represents an important psychological link between domain-general learning resources and mathematics learning. Empirical evidence has consistently linked mathematics self-efficacy with mathematics learning behavior and academic performance. Using a Japanese PISA 2022 sample, Shimizu (2025) found that mathematics self-efficacy was positively associated with behavioral engagement and mathematics achievement and negatively associated with mathematics anxiety. Longitudinal evidence likewise indicates reciprocal associations between mathematics self-efficacy and mathematics achievement over time (Du et al., 2021).

On the other hand, mathematics anxiety reflects tension and worry when students encounter mathematics tasks, the possibility of failure, or evaluative situations. Control–value theory proposes that anxiety is particularly likely when students place high value on an outcome but perceive insufficient control over it (Pekrun, 2006). A substantial body of evidence also shows a robust association between mathematics anxiety and lower mathematics achievement, while self-efficacy and anxiety are dynamically related across development (Barroso et al., 2021; Du et al., 2021; Živković et al., 2023).

Accordingly, the present study conceptualizes mathematics self-efficacy and mathematics anxiety as two proximal psychological states that emerge when domain-general learning resources enter the mathematics microsystem. It further examines whether proactive mathematics learning behavior may provide a behavioral bridge between general engagement and these mathematics-specific psychological states. Accordingly:

*H2*: School-based music participation is serially indirectly associated with mathematics achievement through domain-general learning engagement and its domain-specific recontextualization into mathematics self-efficacy and mathematics anxiety.

### 2.3 Classroom environment and the psychological recontextualization of cross-domain resources

Once domain-general learning engagement enters a specific disciplinary context, its recontextualization into domain-specific psychological states may depend partly on the classroom environment. Self-determination theory emphasizes the role of supportive social environments in satisfying students’ needs for autonomy, competence, and relatedness (Ryan and Deci, 2000), while the prosocial classroom model identifies supportive teacher–student relationships and effective classroom management as complementary conditions for learning (Jennings and Greenberg, 2009). Accordingly, the present study conceptualizes mathematics teacher support and disciplinary climate as the social-support and structural-order conditions of the target classroom, respectively.

Teacher support reflects the academic assistance, emotional care, and competence-related feedback students receive in the classroom and therefore represents an important interpersonal resource. In mathematics, teacher support is closely associated with students’ domain-specific psychological states and subsequent motivation. It has been linked with achievement emotions through control–value appraisals and mathematics self-concept, as well as with subsequent mathematics interest (Chen and Leung, 2023; Hettinger et al., 2023). Teacher behavior may also moderate the association between control–value appraisals and mathematics anxiety, thereby altering the strength with which psychological appraisals are associated with anxiety (Liu et al., 2024). Teacher support may therefore provide an important social condition for the competence appraisals and emotional adaptation through which domain-general resources become recontextualized within mathematics.

In contrast to the strongly interpersonal nature of teacher support, disciplinary climate primarily reflects classroom order and the absence of disruption. A favorable disciplinary climate can reduce interference and help sustain attention to learning tasks, thereby providing a structural basis for continued engagement. Existing research has linked disciplinary climate with mathematics self-efficacy and mathematics self-concept, although these associations vary across student characteristics and contexts (Cheema and Kitsantas, 2014; Ramazan et al., 2023). As an external structural condition, however, disciplinary climate may not have uniformly strong psychological effects. A low-disruption environment can preserve learning opportunities (Wang et al., 2022), but structure itself is not equivalent to emotional support; its psychological meaning may also depend on whether it is experienced as autonomy-supportive rather than controlling (Cheon et al., 2020; Sierens et al., 2009). Longitudinal research has further shown that classroom management is associated with lower mathematics anxiety and boredom, although its associations differ across achievement emotions, whereas teacher support shows broader associations with anxiety, boredom, and enjoyment (Lazarides and Buchholz, 2019). Disciplinary climate may therefore be better understood as a foundational structural condition that supports the use of learning resources but whose association with deeper psychological processes, such as self-efficacy and specific achievement emotions, may be more context- and outcome-dependent.

The role of the classroom environment may also be pathway-specific. Such specificity may arise both from differences between environmental conditions and from differences between psychological outcomes. Teacher support can moderate the association between control–value appraisals and mathematics anxiety (Liu et al., 2024) and can interact differently with individual psychological factors such as academic self-efficacy and mathematics anxiety in relation to classroom engagement (Zhou et al., 2024). These findings suggest that classroom environments need not influence all domain-specific psychological processes uniformly; the direction and strength of moderation may depend on both the environmental factor and the psychological pathway. Given the stronger existing evidence for moderation by teacher support, whereas the psychological role of disciplinary climate appears more context- and outcome-dependent, the present study formulates a theoretical hypothesis for teacher support but retains disciplinary climate as an exploratory research question. Accordingly:

*H3a*: The moderating role of mathematics teacher support in the association between domain-general learning engagement and mathematics-specific psychological states is pathway-specific, differing between the mathematics self-efficacy and mathematics anxiety pathways.

## 3 Method

### 3.1 Data source and sample

This study used publicly available secondary, cross-sectional data from the 2022 Programme for International Student Assessment (PISA), administered by the Organisation for Economic Co-operation and Development (OECD). PISA uses a two-stage school-and-student sampling design targeting approximately 15-year-old students, and mathematics was the major assessment domain in PISA 2022 (OECD, 2023, 2024c). The present analyses used the Korean student database and student questionnaire. No personally identifying information was available in the public-use data.

The Korean student file contained 6,454 students from 186 schools. For the school-based music participation item, the response indicating that the activity was not available at school was excluded from the frequency scale because it reflected availability rather than participation frequency. This resulted in 5,914 students with valid music-participation data. Because some PISA questionnaire blocks were matrix sampled, effective sample sizes varied across measures. RQ1 therefore used outcome-specific analytic samples, whereas RQ2 and RQ3 relied on six high-coverage core engagement indicators. The RQ2 sample comprised 5,136 students from 185 schools, and the RQ3 sample comprised 5,132 students from 185 schools.

### 3.2 Variable extraction and measures

Korean records were extracted from the official PISA 2022 student SPSS file (CY08MSP_STU_QQ.Q.sav). Variables retained for analysis included student and school identifiers, the final student weight, all 80 student replicate weights, the 10 mathematics plausible values, school-based music participation, engagement and learning-strategy measures, mathematics self-efficacy and anxiety, classroom-environment indices, and demographic and educational covariates (see Table 1). OECD special missing-value codes were recoded as missing. Item wording, scoring direction, official scale construction, and matrix-sampling characteristics were checked against the PISA questionnaires and technical documentation (OECD, 2024a, 2024b, 2024c).

**Table 1:** Study measures and PISA variables.

Construct	PISA variable(s)	Operationalization
School-based music participation	ST337Q03JA	Five-category participation frequency
Behavioral engagement	PERSEVAGR; ST062Q01TA–Q03TA	Perseverance and reverse-coded attendance
Cognitive engagement	CURIOAGR; ST303Q01JA, ST303Q03JA	Curiosity and perspective-taking
Metacognitive monitoring	ST309Q04JA; ST309Q05JA	Error and homework checking; supplementary
Emotional–motivational engagement	BELONG; ST268Q07JA–Q09JA	School belonging and achievement motivation
Mathematics-specific learning	MATHPERS	Proactive mathematics study behavior
Mathematics psychological variables	MATHEFF; ANXMAT	Self-efficacy and anxiety
Mathematics achievement	PV1MATH–PV10MATH	Ten plausible values
Classroom environment	TEACHSUP; DISCLIM	Teacher support and disciplinary climate
Covariates	Sex, ESCS, AGE, GRADE, IMMIG, REPEAT	Demographic and educational background
Survey design	W_FSTUWT; W_FSTURWT1–80; CNTSCHID	Final weight, replicate weights, school ID

Measure selection was theory-driven rather than based on observed statistical associations. Official OECD indices were retained when they closely matched the intended construct, whereas researcher-constructed composites were used only when no corresponding published index captured the construct. All derived measures were coded so that higher scores indicated higher levels of the focal construct.

#### 3.2.1 School-based music participation

School-based music participation was measured with ST337Q03JA, which assesses how frequently students participate in music classes or activities at school, including activities such as chorus or band. Responses ranged from 1 (never or almost never) to 5 (every day or almost every day). The “not available at school” response was treated as missing because it represented institutional opportunity rather than actual participation frequency (OECD, 2024a, 2024c). The ordered five-category measure was used in the primary analyses, with a contrast between at least weekly and less-than-weekly participation examined in sensitivity analyses.

#### 3.2.2 Domain-general learning engagement

Following the multidimensional framework proposed by Fredricks et al. (2004), domain-general learning engagement was operationalized across behavioral, cognitive, and emotional–motivational dimensions. Because PISA 2022 did not administer the original Fredricks engagement scale, these variables were mapped onto the theoretical dimensions according to their conceptual content and should therefore be interpreted as PISA-based proxies for multidimensional engagement rather than direct measures of the original scale.

Behavioral engagement was represented by perseverance (PERSEVAGR) and attendance engagement. Behavioral engagement emphasizes sustained participation, effort, and persistence (Fredricks et al., 2004). PERSEVAGR captures students’ tendency to persist with tasks and maintain effort in the face of difficulty. Attendance engagement was derived from ST062Q01TA–Q03TA, covering whole-day absence, class skipping, and lateness. Items were reverse-coded and averaged so that higher scores indicated more regular participation. The two indicators therefore captured complementary aspects of sustained effort and continuity of participation.

Cognitive engagement was represented by curiosity (CURIOAGR) and perspective-taking. CURIOAGR reflects interest in acquiring knowledge, understanding new information, and intellectual exploration. Perspective-taking was constructed from ST303Q01JA and ST303Q03JA and reflects consideration of alternative viewpoints and processing issues from multiple perspectives (OECD, 2024c). These measures were treated as engagement-related cognitive resources reflecting active exploration and cognitive processing rather than as direct indicators of executive function or mathematics-specific learning strategies. Because ST303 was matrix sampled, the primary score used available-item means, with a complete-response specification examined for robustness.

Emotional–motivational engagement was represented by school belonging (BELONG) and general achievement motivation. BELONG is an official PISA index reflecting students’ social and emotional connection to the school environment. General achievement motivation was constructed from ST268Q07JA–Q09JA, which assess students’ desire to perform well in mathematics, test language, and science. Because the composite included mathematics, a sensitivity analysis reconstructed achievement motivation using only test-language and science items to examine whether the findings were driven by mathematics content embedded in the predictor.

Two metacognitive-monitoring items, ST309Q04JA (checking for mistakes) and ST309Q05JA (checking homework before submission), were examined as supplementary indicators of control and self-monitoring strategies (OECD, 2024c; Schraw et al., 2006). Because matrix sampling substantially reduced their effective sample sizes, these indicators were analysed separately rather than included in the six-indicator core engagement set.

#### 3.2.3 Mathematics-specific learning and psychological variables

MATHPERS, the official PISA proactive mathematics study-behavior index, was treated as a mathematics-specific self-regulated learning measure rather than as part of domain-general engagement. Because its content explicitly refers to effort, persistence, task initiation, and participation in mathematics learning, it was positioned as a mathematics-specific learning behavior following the entry of domain-general resources into the receiving subject context (OECD, 2024c).

Mathematics self-efficacy was assessed with MATHEFF and mathematics anxiety with ANXMAT. MATHEFF reflects students’ confidence in completing concrete mathematics tasks, whereas higher ANXMAT scores indicate greater worry, tension, helplessness, and fear of failure in mathematics (OECD, 2024c). The two variables were modelled as parallel mathematics-specific psychological outcomes, without imposing a temporal ordering between them.

Mathematics achievement was represented by PV1MATH–PV10MATH. The 10 plausible values were analysed separately and pooled rather than averaged into a single student score.

#### 3.2.4 Mathematics classroom environment

RQ3 examined perceived mathematics teacher support (TEACHSUP) and disciplinary climate (DISCLIM). TEACHSUP captures perceived support and assistance from the mathematics teacher, whereas DISCLIM reflects classroom order and the extent of disruptive conditions during mathematics lessons. Higher scores represent greater teacher support and a more favorable disciplinary climate, respectively (OECD, 2024c).

#### 3.2.5 Covariates

All adjusted models included sex, ESCS, age, relative grade, immigrant background, and grade repetition. These variables capture major demographic, socioeconomic, and schooling-position differences while avoiding adjustment for learning and psychological variables that could plausibly lie on the proposed cross-domain pathway. The same covariate set was used across RQ1–RQ3 to facilitate comparability.

### 3.3 Measurement quality

For researcher-constructed multi-item measures, internal consistency and inter-item associations were examined in the Korean sample. Attendance engagement showed Cronbach’s α = 0.433, with weighted inter-item correlations ranging from *r* = 0.210 to 0.620 (mean *r* ≈ 0.353). Because the three items capture related but non-interchangeable behaviors—whole-day absence, class skipping, and lateness—the composite was interpreted as a complementary behavioral index rather than as a narrow unidimensional scale. Perspective-taking showed a weighted inter-item correlation of *r* = 0.512 between its two items (α = 0.656). The three-item general achievement-motivation composite showed high internal consistency, Cronbach’s α = 0.912, with weighted inter-item correlations ranging from *r* = 0.730 to 0.814 (mean *r* ≈ 0.773). For official OECD scale indices, including PERSEVAGR, CURIOAGR, BELONG, MATHPERS, MATHEFF, ANXMAT, TEACHSUP, and DISCLIM, the published PISA scale construction and scoring procedures were retained, and their measurement properties were interpreted with reference to the corresponding OECD technical documentation.

The behavioral, cognitive, and emotional–motivational engagement measures used in RQ3 were specified as composites of theoretically complementary rather than interchangeable components. For example, perseverance and attendance engagement jointly represent behavioral engagement but do not constitute parallel indicators of a single narrow latent trait. Cronbach’s α was therefore not used as a criterion for evaluating the internal consistency of these RQ3 composites.

### 3.4 Statistical analysis

All analyses accounted for the PISA complex sampling design. Point estimates used the final student weight (W_FSTUWT), and sampling variance was estimated using all 80 student replicate weights (W_FSTURWT1–W_FSTURWT80) following the PISA balanced repeated replication (BRR)/Fay replication procedure. For analyses involving mathematics achievement, models were estimated separately for PV1MATH–PV10MATH and then pooled across the 10 plausible values. The final coefficient was the mean of the plausible-value-specific estimates, and total variance was calculated as T = \bar U + (1 + 1/M)B.

Where *Uˉ* is the mean sampling variance across plausible values, *B* is the between-plausible-value variance, and *M* = 10. Standard errors and 95% confidence intervals were based on this total variance.

RQ1 used survey-weighted linear regression to examine associations between school-based music participation frequency and the behavioral, cognitive, emotional–motivational, and supplementary metacognitive engagement indicators, adjusting for sex, ESCS, age, grade, immigrant background, and grade repetition. Unstandardized coefficients, standardized *β*s, 95% confidence intervals, and *p* values were reported. Benjamini–Hochberg false-discovery-rate correction was applied to account for multiple testing (Benjamini and Hochberg, 1995).

Sensitivity and robustness analyses included an at-least-weekly participation contrast, categorical frequency models with tests for departure from linearity, common-sample re-estimation of the six core engagement indicators, and alternative specifications of selected engagement measures.

RQ2 was estimated using a survey-weighted recursive path model. The six core domain-general engagement indicators were first modelled as functions of school-based music participation and covariates. MATHPERS was then entered as a mathematics-specific learning-behavior bridge, followed by mathematics self-efficacy (MATHEFF) and mathematics anxiety (ANXMAT), which were modelled in parallel. Mathematics achievement was subsequently regressed on music participation, engagement, MATHPERS, MATHEFF, ANXMAT, and the covariates.

Specific serial indirect associations linking music participation to mathematics achievement through engagement and mathematics-specific learning and psychological processes were estimated. Benjamini–Hochberg false-discovery-rate (BH-FDR) correction was applied to the prespecified specific indirect associations, and effects were additionally summarized by engagement domain and by the self-efficacy and anxiety channels.

The RQ3 model retained the structural ordering used in RQ2 and added teacher support, disciplinary climate, and their interactions with the three engagement composites in predicting mathematics self-efficacy and anxiety. Twelve interaction effects were evaluated individually and jointly using replicate-weight Wald tests, with BH-FDR correction applied across the individual tests.

Conditional indirect associations were examined using indices of moderated mediation (Hayes, 2015) and estimated at −1 *SD*, the mean, and +1 *SD* of the focal moderator while holding the other moderator at its mean. The MATHPERS pathway was not moderated because RQ3 focused specifically on the engagement-to-self-efficacy/anxiety transition.

Because conventional SEM routines do not readily accommodate both PISA replicate weights and 10 mathematics plausible values, the prespecified recursive equations were estimated equation by equation using the survey design. Moderation inference relied primarily on replicate-weight Wald tests and moderated-mediation indices, whereas the comparative fit index (CFI), Tucker–Lewis index (TLI), root mean square error of approximation (RMSEA), and standardized root mean square residual (SRMR) were treated as supplementary diagnostics.

All statistical tests were two-sided with *α* = 0.05. Because PISA 2022 is cross-sectional, the ordering of variables reflects the proposed theoretical structure rather than observed temporal sequencing; indirect effects are therefore interpreted as statistical indirect associations rather than causal mediation effects.

### 3.5 Software

Data preparation and statistical analyses were conducted in Python 3.13.5 using pandas 2.2.3, NumPy 2.3.5, SciPy 1.17.0, and statsmodels 0.14.6. Custom functions were used to implement PISA replicate-weight variance estimation, plausible-value pooling, indirect-effect calculations, Wald tests, and covariance-based fit indices for the composite structural model. To assess computational validity, representative models were re-estimated using direct calls to the standard statsmodels weighted least-squares implementation, independently of the custom analysis wrappers. The resulting coefficients and replicate-weight standard errors agreed with the custom implementation to numerical precision. Plausible-value pooling was also independently verified using the OECD pooling formula applied to estimates and BRR sampling variances from each of the 10 plausible values. Because no single standard Python routine jointly implements PISA replicate weighting, plausible-value pooling, and the moderated indirect-effect calculations used here, validation was conducted component by component. No discrepancies affecting the reported results were identified. Quality-control procedures were used to verify analytic sample sizes, coding direction, missingness, replicate-weight calculations, and plausible-value variance decomposition.

## 4 Results

### 4.1 School-based music participation and domain-general learning engagement

The Korean PISA 2022 sample comprised 6,454 students from 186 schools, of whom 5,914 had valid data on school-based music participation. Population-weighted estimates indicated that 58.3% of students participated in school-based music classes or activities at least once a week. All models controlled for gender, ESCS, age, grade, immigrant background, and grade repetition, with standard errors estimated using the final student weight and 80 replicate weights.

The primary analyses showed that music participation frequency was significantly and positively associated with all eight learning-engagement indicators, and all associations remained significant after BH-FD, with standardized coefficients ranging from *β* = 0.034 to 0.083 (see Table 2 and Figure 1). For behavioral engagement, perseverance was associated with music participation at *B* = 0.025, *SE* = 0.010, *β* = 0.034, *p* = 0.015, *q* = 0.015, and attendance engagement at *B* = 0.011, *SE* = 0.004, *β* = 0.060, *p* = 0.009, *q* = 0.011. For cognitive engagement, curiosity showed an association of *B* = 0.059, *SE* = 0.016, *β* = 0.072, *p* < 0.001, *q* < 0.001, and perspective-taking of *B* = 0.039, *SE* = 0.010, *β* = 0.062, *p* < 0.001, *q* < 0.001. Both supplementary metacognitive indicators were also significant: error checking, *B* = 0.035, *SE* = 0.013, *β* = 0.060, *p* = 0.007, *q* = 0.009, and homework checking, *B* = 0.063, *SE* = 0.017, *β* = 0.083, *p* < 0.001, *q* < 0.001. For emotional–motivational engagement, school belonging was associated with music participation at *B* = 0.042, *SE* = 0.009, *β* = 0.056, *p* < 0.001, *q* < 0.001, and general achievement motivation at *B* = 0.039, *SE* = 0.009, *β* = 0.074, *p* < 0.001, *q* < 0.001.

**Table 2:** Music participation and learning engagement.

Engagement indicator	*N*	*B* (*SE*)	95% *CI*	*β*	*p*	BH-FDR *q*
Perseverance	5,826	0.025 (0.010)	[0.005, 0.046]	0.034	0.015	0.015
Attendance engagement	5,856	0.011 (0.004)	[0.003, 0.019]	0.060	0.009	0.011
Curiosity	5,822	0.059 (0.016)	[0.026, 0.091]	0.072	< 0.001	< 0.001
Perspective-taking	5,240	0.039 (0.010)	[0.020, 0.058]	0.062	< 0.001	< 0.001
Metacognitive error checking†	2,938	0.035 (0.013)	[0.009, 0.060]	0.060	0.007	0.009
Metacognitive homework checking†	2,880	0.063 (0.017)	[0.029, 0.096]	0.083	< 0.001	< 0.001
School belonging	5,870	0.042 (0.009)	[0.024, 0.059]	0.056	< 0.001	< 0.001
General achievement motivation	5,869	0.039 (0.009)	[0.021, 0.058]	0.074	< 0.001	< 0.001

B represents the change in the outcome associated with a one-category increase in school-based music participation frequency. All models adjusted for gender, ESCS, age, grade, immigrant background, and grade repetition. † ST309 metacognitive items were matrix sampled, resulting in smaller outcome-specific analytic samples.

**Figure 1:**
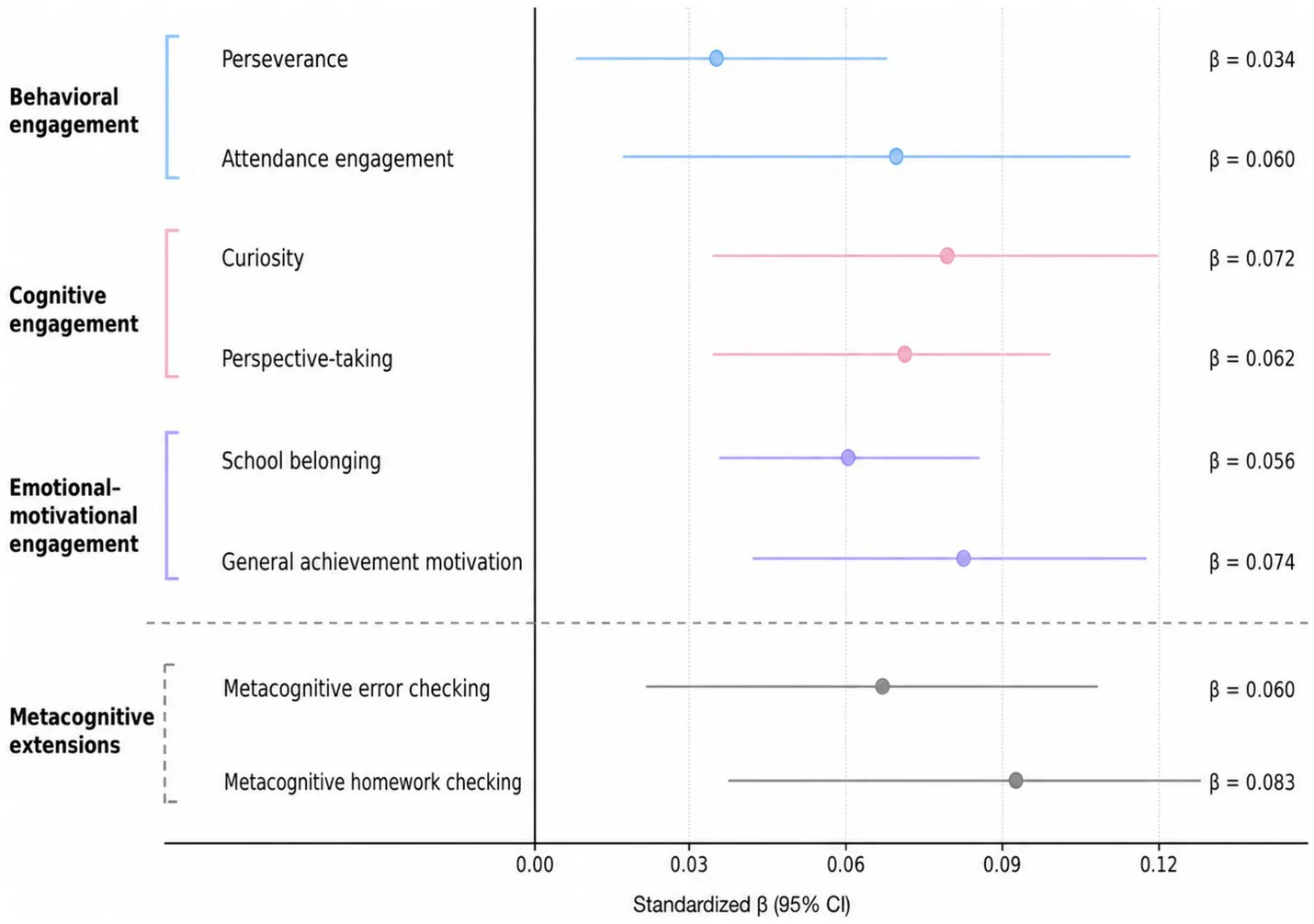
Music participation and learning engagement.

Sensitivity and robustness analyses generally supported this multidimensional pattern. When music participation was recoded as participation at least once per week, all eight associations remained significant. Tests of nonlinearity, however, remained significant after BH-FDR only for curiosity and general achievement motivation. In the common-sample analysis, five of the six core indicators remained significant, whereas perseverance weakened to marginal significance, *B* = 0.023, *p* = 0.054. When the sample was further restricted to students who completed both ST303 items, the association with perspective-taking was no longer significant, *N* = 2,077, *B* = 0.027, 95% CI [−0.011, 0.066], *p* = 0.169. However, the single-item indicator of critical open-mindedness remained significant, *B* = 0.033, *p* = 0.020.

Overall, more frequent school-based music participation was associated with small but generally consistent positive differences in behavioral, cognitive, and emotional–motivational engagement. Although the associations for perseverance and perspective-taking weakened in some robustness analyses, the primary pattern remained stable across alternative participation coding, common-sample analyses, and most alternative measurement specifications. H1 was therefore broadly supported, although this does not imply that every individual engagement indicator was equally robust across all analytical specifications.

### 4.2 Domain-specific psychological pathways linking music participation and mathematics achievement

RQ2 was examined using 5,136 students from 185 schools. The analysis first tested whether school-based music participation was indirectly associated with mathematics achievement through domain-general learning engagement and mathematics-specific psychological states. Before the intermediate variables were introduced, each one-category increase in music participation frequency was associated with a 5.93-point increase in PISA mathematics achievement, *SE* = 1.76, 95% CI [2.49, 9.37], *p* < 0.001. After domain-general learning engagement, mathematics self-efficacy, and mathematics anxiety were included, the residual direct association between music participation and mathematics achievement was substantially attenuated.

For the core psychological recontextualization pathways, domain-general learning engagement showed a significant indirect association with mathematics achievement through mathematics self-efficacy, *B* = 0.498, *SE* = 0.134, 95% CI [0.236, 0.760], *p* < 0.001 (see Table 3 and Figure 2). By contrast, the overall indirect association through mathematics anxiety was not significant, *B* = −0.034, *SE* = 0.030, 95% CI [−0.093, 0.024], *p* = 0.254. The aggregated indirect associations for behavioral, cognitive, and emotional–motivational engagement were all significant, with emotional–motivational engagement showing the largest overall association. H2 was therefore broadly supported, although the cross-domain recontextualization pattern was concentrated primarily in the mathematics self-efficacy pathway rather than reflecting a general reduction in mathematics anxiety.

**Table 3:** Main indirect associations with mathematics achievement.

Path	Indirect *B*	*SE*	95% *CI*	*p*
Engagement → self-efficacy → mathematics	0.498	0.134	[0.236, 0.760]	<0.001
Engagement → anxiety → mathematics	−0.034	0.030	[−0.093, 0.024]	0.254
Engagement → MATHPERS → self-efficacy → mathematics	0.435	0.103	[0.234, 0.637]	<0.001
Engagement → MATHPERS → anxiety → mathematics	0.047	0.014	[0.019, 0.075]	0.001
Behavioral engagement	0.228	0.104	[0.024, 0.431]	0.028
Cognitive engagement	0.232	0.087	[0.061, 0.402]	0.008
Emotional–motivational engagement	0.487	0.141	[0.210, 0.763]	<0.001
All focal psychological-conversion paths	0.946	0.235	[0.486, 1.407]	<0.001

**Figure 2:**
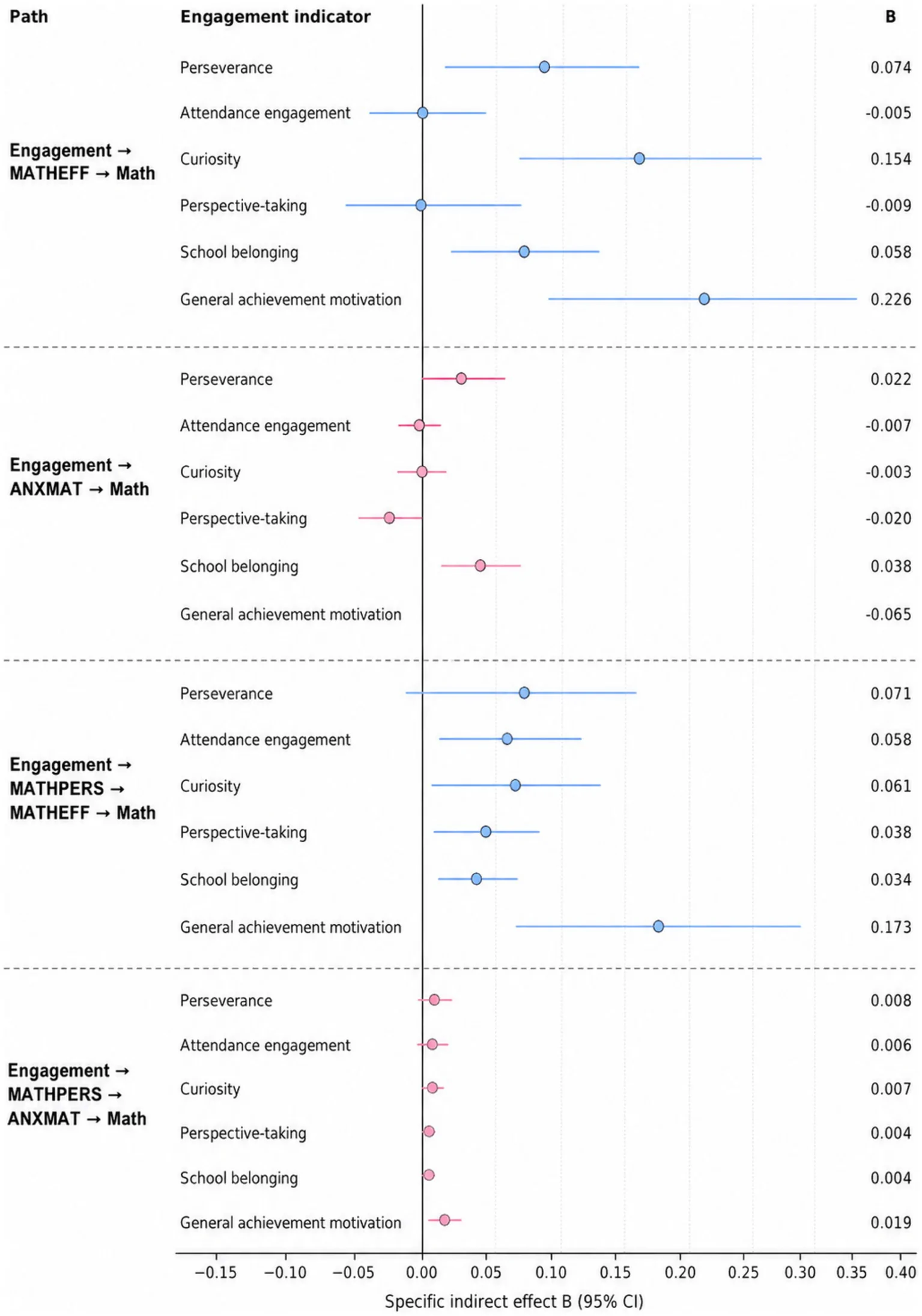
Specific indirect paths.

As an exploratory extension, MATHPERS was examined as a possible behavioral bridge between domain-general engagement and mathematics-specific psychological states. All six core engagement indicators were positively associated with MATHPERS (all *p*s < 0.001), which was in turn associated with higher mathematics self-efficacy (*B* = 0.436, *p* < 0.001) and lower mathematics anxiety (*B* = −0.218, *p* < 0.001). Serial indirect associations with mathematics achievement were significant through MATHPERS and self-efficacy (*B* = 0.435, *p* < 0.001) and through MATHPERS and anxiety (*B* = 0.047, *p* = 0.001), whereas the residual association between MATHPERS and achievement was nonsignificant (*B* = 3.33, *p* = 0.190). These findings provide exploratory evidence that proactive mathematics learning behavior may help connect domain-general engagement with mathematics-specific psychological processes, rather than constituting a necessary component of H2. Sensitivity analyses generally supported the core findings. When music participation was recoded as participation at least once per week, the overall core indirect association remained significant. Re-estimating the model among students with complete responses to the ST303 items also yielded a significant overall core indirect association, *N* = 2,030, *B* = 0.821, *p* = 0.010. Results were similarly stable when general achievement motivation was reconstructed after excluding its mathematics component, *N* = 5,134, *B* = 0.914, *p* < 0.001. Thus, although the individual perspective-taking indicator weakened in the complete-response sensitivity analysis for RQ1, the overall domain-specific recontextualization pattern in RQ2 did not depend on that particular scoring specification.

Overall, H2 was supported. Domain-general learning engagement was indirectly associated with mathematics achievement primarily through mathematics self-efficacy, whereas the overall mathematics-anxiety pathway was not significant. Exploratory MATHPERS analyses further suggested a possible behavioral bridge in this domain-specific recontextualization process.

### 4.3 Conditional role of the mathematics classroom environment

RQ3 was examined using 5,132 students from 185 schools and tested whether mathematics teacher support and disciplinary climate moderated the associations between the three dimensions of domain-general learning engagement and mathematics self-efficacy and anxiety. Behavioral, cognitive, and emotional–motivational engagement were represented by composite indicators, and the model simultaneously included teacher support, disciplinary climate, and the 12 interaction terms formed between the two classroom conditions and the three engagement dimensions across the two psychological outcomes.

First, the omnibus joint test of the 12 interaction terms was significant, Wald χ^2^(12) = 44.63, *p* < 0.001, indicating that the classroom-environment interactions collectively contained significant moderation information (see Table 4; Figure 3). This result, however, does not imply that all 12 individual interactions were significant. Joint tests by psychological outcome further showed that the combined moderation of teacher support on the associations between the three engagement dimensions and mathematics anxiety was significant, Wald χ^2^(3) = 25.56, *p* < 0.001, whereas its combined moderation of mathematics self-efficacy was not significant, *p* = 0.126. Thus, the moderation signal for teacher support was concentrated primarily in the mathematics-anxiety pathway rather than the mathematics-self-efficacy pathway.

**Table 4:** Main classroom-environment moderation results.

Test	Estimate	95% *CI*	*p*	FDR *q*
All 12 interactions	Wald *χ^2^*(12) = 44.63	—	<0.001	—
Teacher support × engagement → anxiety	Wald *χ^2^*(3) = 25.56	—	<0.001	<0.001
Teacher support × engagement → self-efficacy	—	—	0.126	—
Emotional–motivational engagement × teacher support → anxiety	*B* = −0.068	[−0.105, −0.030]	<0.001	0.006
Teacher-support moderated-mediation index	−0.035	[−0.114, 0.045]	0.391	—
Disciplinary-climate moderated-mediation index	0.022	[−0.073, 0.118]	0.645	—

**Figure 3:**
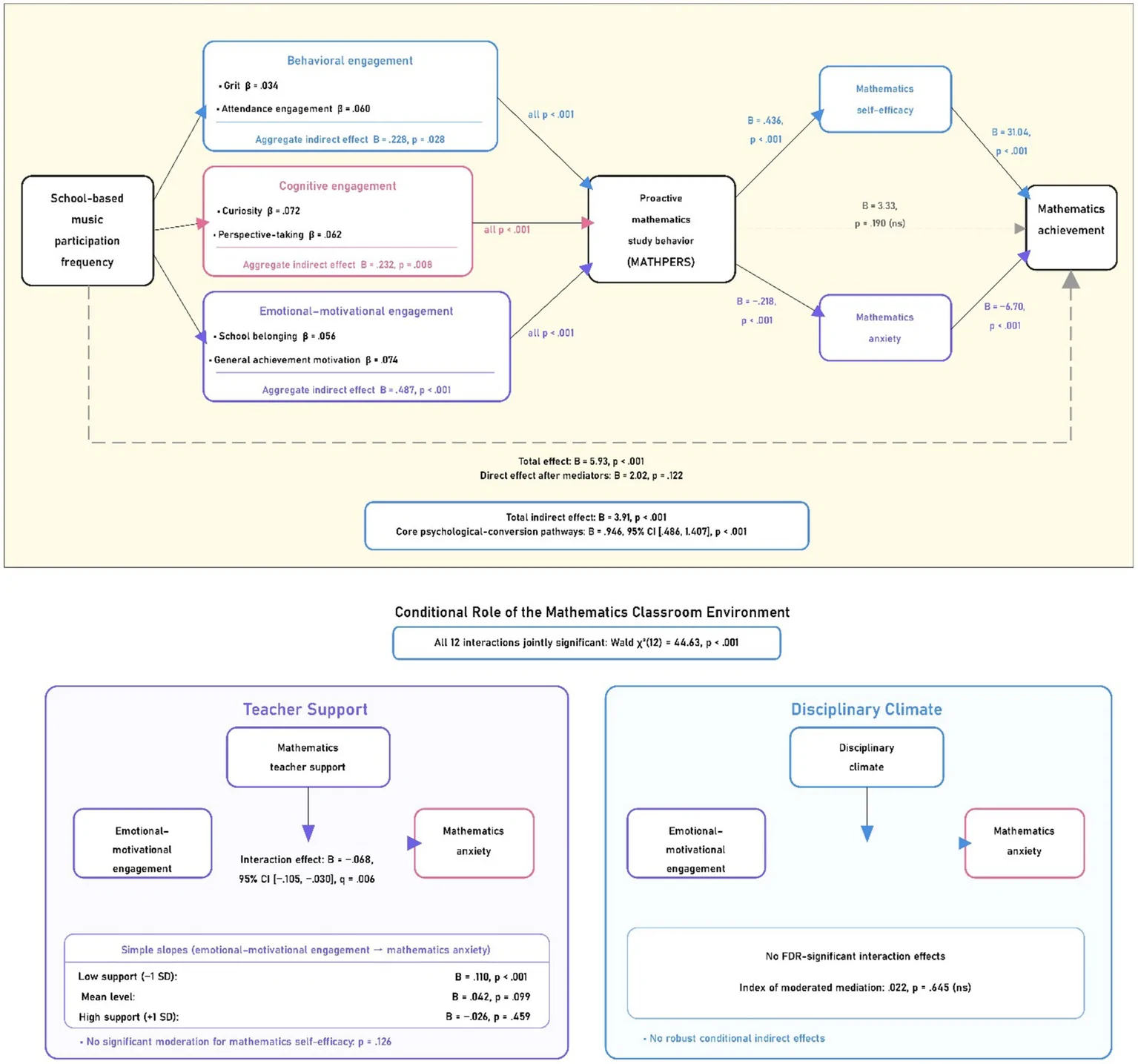
Moderated path model of classroom environment.

To further localize this overall moderation signal, the 12 individual interactions were decomposed exploratorily and subjected to BH-FDR. After correction, only the interaction between emotional–motivational engagement and teacher support in relation to mathematics anxiety remained significant, *B* = −0.068, *SE* = 0.019, 95% CI [−0.105, −0.030], *p* < 0.001, *q* = 0.006. None of the individual interactions involving disciplinary climate survived BH-FDR. This specific interaction should therefore be interpreted as an exploratory localization of the broader moderation signal rather than as a separately prespecified hypothesis test.

Simple-slope analyses further showed that, at low teacher support (−1 *SD*), emotional–motivational engagement was positively associated with mathematics anxiety, *B* = 0.110, *SE* = 0.029, 95% CI [0.053, 0.166], *p* < 0.001. At the mean level of teacher support, the association was not significant, *B* = 0.042, *SE* = 0.025, *p* = 0.099. At high teacher support (+1 *SD*), the association was close to zero, *B* = −0.026, *SE* = 0.035, *p* = 0.459. These results indicate that teacher support primarily attenuated the association between higher emotional–motivational engagement and greater mathematics anxiety under low-support conditions, rather than uniformly strengthening the recontextualization of domain-general engagement into mathematics-specific psychological states.

Further conditional indirect-effect analyses provided no robust evidence that the classroom environment altered the overall cross-domain indirect association. The overall index of moderated mediation for teacher support was −0.035, *SE* = 0.041, 95% CI [−0.114, 0.045], *p* = 0.391, whereas the corresponding index for disciplinary climate was 0.022, *SE* = 0.049, 95% CI [−0.073, 0.118], *p* = 0.645. The pathway-specific indices of moderated mediation through mathematics self-efficacy and mathematics anxiety were also nonsignificant for both classroom conditions.

At the same time, the core indirect association between music participation and mathematics achievement remained significant across levels of both classroom conditions. At −1 *SD*, the mean, and +1 *SD* of teacher support, the conditional indirect associations were *B* = 1.084, 1.050, and 1.015, respectively. The corresponding estimates for disciplinary climate were *B* = 1.027, 1.050, and 1.072, respectively, all *p*s < 0.001. These findings suggest that classroom conditions may selectively moderate localized psychological pathways but did not provide evidence of changes in the overall strength of the cross-domain indirect association.

Overall, the findings for RQ3a indicated a localized moderation signal for teacher support, concentrated in the mathematics-anxiety pathway rather than the mathematics-self-efficacy pathway. Further exploratory decomposition localized this signal to the interaction between emotional–motivational engagement and teacher support. For RQ3b, disciplinary climate did not show robust moderation after correction for multiple testing. Importantly, neither teacher support nor disciplinary climate significantly altered the overall cross-domain indirect association, indicating that the observed teacher-support moderation was localized rather than evidence of a general moderated indirect effect.

## 5 Discussion and conclusion

The present study examined cross-domain associations between school-based music participation and mathematics learning from the perspective of connections among disciplinary contexts within the school. The findings broadly supported H1 and H2 and provided partial support for H3a, with teacher-support moderation confined to a localized mathematics-anxiety pathway rather than the overall cross-domain indirect association. More frequent school-based music participation was associated with small positive differences across behavioral, cognitive, and emotional–motivational engagement, although the robustness of some individual indicators varied across sensitivity analyses. These domain-general learning resources were further indirectly associated with mathematics achievement through mathematics-specific psychological states, with mathematics self-efficacy emerging as the primary psychological pathway. Exploratory analyses further suggested that proactive mathematics learning behavior may contribute to this process of domain-specific recontextualization. Classroom moderation, by contrast, was concentrated in a specific anxiety pathway and did not appear to alter the overall cross-domain indirect association.

### 5.1 From “far transfer” in music to cross-domain resource formation

School-based music participation was not associated with a single isolated capacity but with a set of domain-general learning resources spanning behavioral, cognitive, and emotional–motivational engagement. This pattern is broadly consistent with previous evidence linking music participation with general cognition, school connectedness, and positive youth development (Ilari and Cho, 2023; Román-Caballero et al., 2022), while the present study integrates these relatively dispersed outcomes within a multidimensional learning-engagement structure.

This multidimensional pattern may reflect the simultaneous demands that music learning places on sustained engagement, feedback regulation, active exploration, and social participation. Music practice itself requires continuing motivation and self-regulation, while musical activities also involve expression, exploration, and interaction (Evans, 2023), making them potentially relevant to several forms of learning resources.

The strength of these associations was not uniform, however. Curiosity and general achievement motivation showed comparatively stronger associations with music participation, whereas perseverance showed a weaker association; the former two also displayed some evidence of nonlinearity, indicating that increasing participation frequency does not necessarily correspond to linear increases across all resources. Exploration, goal pursuit, and continuous feedback are relatively close to the structure of musical activity and may therefore be more readily associated with curiosity and learning motivation. By contrast, attendance is shaped by individual, family, school, and broader contextual influences (Hidajat et al., 2026), potentially limiting its independent association with music participation. Some behavioral and cognitive indicators also weakened under alternative sample or measurement specifications, further suggesting that the findings are better understood as a small but multidimensional overall engagement pattern rather than as equally robust associations for every individual indicator. At the same time, the standardized associations were small (β = 0.034–0.083), and statistical significance should not be taken to imply substantial educational effects in practice (Funder and Ozer, 2019). The present findings therefore speak more to the consistency of the multidimensional association pattern than to the magnitude of its real-world impact.

### 5.2 Domain-specific recontextualization and classroom conditions

H2 further indicated that the association between domain-general resources and mathematics achievement was primarily linked to more proximal mathematics-specific psychological states. Mathematics self-efficacy represented a comparatively stable psychological pathway, whereas mathematics anxiety showed more heterogeneous and partly countervailing pathways.

This distinction may reflect different functions of motivational–emotional and behavioral–cognitive resources during domain-specific recontextualization. Self-efficacy is strongly task-specific and reflects individuals’ judgments about whether they can organize and successfully perform particular actions (Bandura, 1997). General achievement motivation is closely related to goal pursuit and sustained effort and may therefore be more readily associated with competence beliefs and learning engagement. Previous evidence likewise links higher achievement motivation with stronger general self-efficacy and indicates reciprocal relations between motivation and cognitive engagement in mathematics learning (Li et al., 2023; Zhang et al., 2023). School belonging, by contrast, reflects the social–emotional experience of being accepted, supported, and integrated into the school community and is more commonly associated with learning engagement, motivation, and broader psychological adjustment than with domain-specific competence judgments directly (Allen et al., 2024; St-Amand et al., 2021). Attendance primarily reflects continuity of participation, whereas perspective-taking reflects the cognitive processing of different viewpoints and information. These resources are therefore more closely aligned with behavioral participation and cognitive processing than with mathematics competence judgments themselves, and their relevance may need to become further recontextualized through actual mathematics tasks.

Mathematics anxiety showed greater pathway heterogeneity. School belonging may provide a broader social–emotional basis for psychological adjustment through feelings of connection and support (Allen et al., 2024), whereas different achievement-goal configurations may be associated with different levels of mathematics anxiety and self-efficacy (Chan and Liem, 2023). According to control–value theory, anxiety becomes more likely when students attach high value to an outcome but perceive insufficient control over it (Pekrun, 2006; Putwain and Wood, 2023). Achievement motivation may therefore be better understood here as a resource with dual motivational implications, relating both to task engagement and to evaluative sensitivity, rather than as a uniformly protective or risk-related factor.

Beyond the core H2 analysis, exploratory findings suggested that proactive mathematics learning behavior (MATHPERS) may serve as a behavioral bridge when general learning resources enter the mathematics context. Its potential role lies in translating relatively general learning tendencies into concrete task participation, strategy use, and feedback experiences, which may in turn be associated with mathematics self-efficacy and anxiety, rather than merely increasing the quantity of learning behavior and thereby corresponding directly to higher achievement. This interpretation is consistent with views of self-regulated learning that emphasize interactions among cognition, behavior, and motivation (Theobald, 2021). Because this finding represents an exploratory extension of the mechanism, however, it is best used to illuminate a possible process of domain-specific recontextualization rather than treated as a necessary component of H2.

RQ3 further revealed a localized moderation pattern involving teacher support. The omnibus tests indicated that the moderating signal of teacher support was concentrated in mathematics anxiety rather than mathematics self-efficacy, providing partial support for H3a. Exploratory decomposition further showed that, after BH-FDR, only the interaction between emotional–motivational engagement and teacher support in relation to mathematics anxiety remained statistically significant. Emotional support, academic assistance, and competence-related feedback from teachers may strengthen students’ perceived control over mathematics tasks and thereby attenuate the unfavorable association between high engagement and high anxiety (Chen and Leung, 2023; Liu et al., 2024).

By contrast, disciplinary climate did not show robust moderation, suggesting that it may primarily provide structural conditions for maintaining classroom order and reducing disruption rather than directly altering students’ appraisals of competence and pressure. Moreover, neither classroom condition significantly changed the overall cross-domain indirect association. The classroom environment may therefore exert more localized effects on particular psychological pathways rather than functioning as a uniform condition that determines whether cross-domain resource recontextualization occurs.

Overall, mathematics provided not only a clear final achievement indicator but also an opportunity to observe how domain-general resources become psychologically recontextualized within a specific academic domain. The findings therefore support an interpretation in which connections between school subjects need not involve direct transfer between endpoint abilities; instead, general learning resources may acquire new domain-specific meaning within a target subject and ultimately become manifested in observable academic performance.

### 5.3 Limitations and future research

Although the present study used the large Korean PISA 2022 sample and incorporated complex sampling weights, replicate weights, and mathematics plausible values to improve the representativeness and robustness of the estimates, several boundaries require further examination.

First, PISA 2022 is inherently cross-sectional. The ordering of variables in the present study therefore reflects the proposed theoretical structure rather than observed temporal sequencing. The study can identify statistical associations and indirect associations among music participation, domain-general learning resources, mathematics-specific psychological states, and achievement, but it cannot establish causal direction. Accordingly, the proposed sequence of resource formation, mobilisation, and recontextualisation should not be interpreted as an empirically observed developmental process. Future studies could use longitudinal pre–post designs, curriculum-based follow-up studies, or quasi-experimental designs to investigate the temporal process of resource formation and domain-specific recontextualization more directly.

Second, a major advantage of PISA is that music participation, learning engagement, classroom conditions, and mathematics outcomes can be examined within the same large-scale framework. However, these indicators were not specifically designed for the cross-domain resource model proposed here. Behavioral, cognitive, and emotional–motivational engagement were therefore theoretically mapped onto available PISA indicators, and some constructs were represented by short scales or proxy measures with limited construct coverage. These psychometric constraints mean that the indicators should be interpreted as approximations of the intended resource domains rather than as comprehensive measures of the underlying constructs. Future research could combine dedicated multidimensional engagement scales with classroom observation and behavioral data to measure both the resources themselves and their domain-specific recontextualization more precisely.

Third, school-based music participation was represented primarily by participation frequency. This captures the extent of participation but cannot distinguish curriculum content, activity type, instructional quality, or students’ specific roles within musical activities. Because participation was measured with a single broad item, the measure provides only limited coverage of students’ musical experiences. Moreover, more frequent participants may differ in unobserved characteristics, such as prior musical interest, family support, or school opportunities, so residual selection bias cannot be excluded. The present findings are therefore better suited to identifying overall associations between music participation and cross-domain resources than to determining which forms of music education are most strongly associated with such resources. Future research could compare activities with different task structures, including choral singing, instrumental learning, composition, and collaborative performance, while simultaneously recording actual curriculum time in music and mathematics to assess potential time costs and cross-domain resource-related benefits more directly.

Finally, mathematics was selected as a representative core domain of adolescent learning and academic development in PISA 2022. However, the cognitive demands, evaluative pressures, and domain-specific psychological processes characteristic of mathematics cannot represent all academic subjects. Future research should therefore replicate the proposed resource-recontextualization mechanism in reading, science, and other learning domains to clarify its cross-domain boundaries and generalizability.

### 5.4 Educational implications and theoretical contributions

The present study suggests that the association between music participation and mathematics learning is not primarily characterized by direct achievement transfer, but may instead involve domain-general learning resources and their subsequent recontextualization within mathematics.

First, decisions about music provision in schools should not depend primarily on whether music can “raise mathematics scores.” Greater attention should also be given to music learning itself and to its potential associations with developmental resources such as sustained engagement, active exploration, self-regulation, and school connectedness. Decisions about arts provision should therefore not rely solely on performance in core academic subjects.

Second, cross-disciplinary education need not be limited to content-level STEAM integration. Different subjects may remain relatively autonomous while still becoming connected through the learning resources carried by students. While maintaining its own disciplinary aims, the music classroom may provide sustained practice, feedback-based revision, active exploration, and reflection that reinforce resources usable across contexts. Mathematics classrooms, in turn, may help students recontextualize these general resources through concrete tasks, strategy guidance, and competence-related feedback, allowing them to become expressed as mathematics-specific learning behaviors and competence beliefs.

From a curriculum-policy perspective, decisions about time allocated to arts education should also extend beyond a simple “crowding-out” logic. Limited curriculum time undoubtedly entails opportunity costs, but arts participation may also be associated with developmental resources that remain relevant across other learning contexts. Curriculum evaluation should therefore consider the disciplinary value of arts education, students’ developmental resources, and actual curriculum-time costs simultaneously rather than expanding or reducing arts provision solely on the basis of whether it directly improves achievement in core subjects. More direct tests of this trade-off will require future studies to record actual time allocations across subjects alongside changes in student resources and longer-term academic development.

Theoretically, the present study makes two main contributions.

First, it proposes and provides preliminary support for a heterogeneous parallel disciplinary microsystems perspective, in which different school subjects are conceptualized as relatively autonomous learning microsystems that can nevertheless become connected through the student. Cross-domain associations can therefore be conceptualized as a process of resource formation–cross-context mobilization–domain-specific recontextualization–academic manifestation, rather than as direct transfer from the outcome of one subject to the outcome of another. Domain-general resources developed or reinforced in one disciplinary context may acquire new domain-specific meaning in another as they encounter different task demands, psychological appraisals, and classroom conditions.

Second, the study moves the cross-domain value of music education beyond the outcome-oriented question of whether it produces distal achievement advantages and toward the examination of learning-development resources and their domain-specific recontextualization mechanisms. The significance of music participation lies not only in whether it is ultimately associated with mathematics achievement, but also in the types of domain-general resources with which it is associated and in how these resources become expressed as domain-specific competence beliefs, emotional states, and learning behaviors after entering another core academic domain, before ultimately becoming associated with academic performance. Mathematics achievement therefore does not define the effect of music education itself; rather, it serves as an observable manifestation of this recontextualization process within one representative core academic domain.

In conclusion, the present study contributes to understanding resource connections among disciplinary microsystems within schools, advances a mechanism-based interpretation of the cross-domain relevance of arts education, and provides theoretical and empirical implications for the allocation and evaluation of arts curricula.

## Data Availability

Publicly available datasets were analyzed in this study. The data analysed in this study are publicly available from the OECD PISA 2022 database (https://www.oecd.org/en/data/datasets/pisa-2022-database.html).
